# Cancer of the Uterus; Compression of the Uterus; Consecutive Hydronephrosis; Suppression of the Urinary Secretion; Nervous Complications

**Published:** 1860-12

**Authors:** M. Aaran

**Affiliations:** Hotel-Dien


ATLANTA

Medical and Surgical Journal.

Vol. VI.] DECEMBER, 1860. [No. 4.

### ORIGINAL COMMUNICATIONS.

### ARTICLE I.

*Cancer of the Uterus; Compression of the Uterus; Consecutive Hydronephrosis; Suppression of the Urinary Secretion; Nervous Complications;* By M. Aran, at the Hotel-Dieu. (*Translated from the Gazette des Hospitaux.*) By Robert Battey, M. D., Rome, Ga.

One of our patients has succumbed in the last stage of cancerous cachexia, after having presented in the latter part of her life some cerebral phenomena, which, although frequent in cancer of the uterus, are, nevertheless, as yet, little understood. I will profit, then, of this occasion, to say to you a few words upon the principal phenomena of cancer of the uterus, and to draw your attention to the most usual terminations of this cruel malady.

This woman, aged 55 years, entered this hospital the 18th of last April, and died the 17th of July. You have all been struck with her aspect of suffering, and with the considerable emaciation that she exhibited. She survived the onset of her malady but four months, and we shall see presently what of significance is to be found in this assertion. Be that as it may, endowed formerly with a strong and robust constitution, she enjoyed habitually good health. She has never been subjected to privation nor grief, nor has she ever suffered destitution. There is no antecedent diathesis in her family. Her catamenia has been regular, and she has been without leucorrhœa from the age of 15 to 47 years. She has had four children. The first accouchment, at the age of 21, was laborious, but not followed by any morbid manifestations upon the part of the uterus or its appendages, and the other three presented nothing remarkable either in their course or their consequences. She has never had any other affection except a fever, the nature of which she could not tell, and a pleurisy at the age of 35, of which she recovered perfectly.

Her health was good up to the epoch of the cessation of the menses, at which time appeared a very abundant leucorrhœa, and the menstruation became irregular. The patient was now subject to uterine hemorrhages which showed themselves sometimes at the menstrual periods, at other times in the intervals. In a word, these hemorrhages presented no regularity, either in their course or their quantity. A symptom important to notice, and which showed itself at this time, was the appearance of some lancinating pains which the patient referred to the womb. Little by little the leucorrhœa diminished, as well as the hemorrhages, which ceased entirely, and the patient experienced no further inconvenience for the space of two years and a half. But, four months ago, the face of things was changed—the appetite diminished, the strength declined day by day, and the emaciation, of which you have been able to see considerable progress, commenced to manifest itself. It had been, however, but seven weeks before the entrance of the patient into the hospital, that acute lancinating pains had begun to be felt in the lower abdomen. These pains, which gradually irradiated themselves into the left thigh, following the direction of the great sciatic nerve, became strong enough not only to impede the walking but also to interrupt sleep. A very important phenomena occurred at the same time. The patient sustained a loss of serous liquid of a brownish color, and exhaling a most horrible fœtid odor. Twice already, in four months, the same symptom has occurred, and you will directly find an explanation for this, when you will have the anatomical specimens before you. Notwithstanding this state of general suffering, the patient had not entirely renounced her *rapports sexuels*, and in consequence she had one day a pretty abundant hemorrhage. The debility increasing still upon her, as well as the pains, she entered one month afterwards into our service.

Recall to yourselves her cachetic aspect, the pale yellow special coloration of her skin, her extreme emaciation, and the feeble condition in which she found herself—a prey to the atrocious pains which filled all the lower abdomen and especially the left side—she was not able to enjoy a moment of repose. At other times the pains irradiated themselves like darts of lightning in all directions, but more particularly along the track of the left great sciatic nerve. She was, moreover, tormented by an obstinate constipation, and an almost incessant desire to void urine, while at the same time there was from the external genitals a horribly fœtid sanious flow.

You know all the diagnostic value that medical men accord to the fœtid vaginal discharges, especially whenever encountered under circumstances analogous to those presented in our patient. This sign is considered by them as almost pathognomonic of cancer of the uterus. Fully recognizing on my part that one should lay much stress upon this symptom, I believe that before making the diagnosis it is necessary to search for other symptoms. In fact, the same fœtid discharges show themselves at certain periods in the evolutions of polypi. In this latter malady, the women, exhausted by the abundant losses of blood, and a prey also to the acute pains, do not fail to present certain traits of a less superficial resemblance to those of a cancer of the uterus.

The presence of gas mixed with this sanious liquid is, especially for some physicians, a certain index of a cancer of the internal cavity of the womb. I ought also to dissipate this error from your minds, for in one case where this sign existed, and of which I have published an account, the cure was complete.

This digression terminated, I will return to our patient—and I say, if, taking account of the former hemorrhages, alternately with the serious leucorrhœal discharges, the lancinating pains seated in the lower abdomen, of the loss of blood which has been determined one month by the coitus, of the considerable emaciation, of the special coachetic taint, and, finally, of this fœtid discharge to which I have drawn your attention—if, I say, recalling all these symptoms, and having presented to the mind the circumstances under which the disease has declared itself, we are able to presume that there exists a cancer of the uterus, the physical signs—that is to say, those that we have furnished us in the various modes of taxis—permit us to pronounce ourselves in perfect security.

In fact, the vagina is humid and bathed in a sanious liquid in which is intermixed the detritus of organic materials. The parieties do not present the regularity and supleness of the normal state. Above, in the upper part of the vagina where it embraces the neck of the uterus, are felt bumps, nodules of induration, vigetating inequalities projecting towards the interior, which give rise to a notable diminution of the calibre of this canal. Beyond these projections of indurated tissue, is felt higher up and at a point far removed from the vulva, the neck of the uterus completely fixed, immovable as if enclosed in the midst of a substance hard and resistant to the touch. It presents the form of a stump (moignon) bristling with nodules, elevated projections bleeding easily, of a variable consistence, and which are continuous with the inequalities which we have found in the upper part of the vagina.

By the rectum, of which the calibre is notably reduced by the pressure of a tumor situated in front of it, we recognize the posterior face of the uterus, which is rendered completely immovable by the adherences and false membranes which fill up the entire pelvic cavity. The direction of the rectum appears to be a little deviated, it seems to be carried a little forward and soldered to the uterus, and its parieties have lost their natural elasticity, doubtless by the thickening of the cellular tissue of the pelvis, invaded gradually by the tumor, of which the point of origin has been the neck of the uterus.

Do you not understand now why the patient, habitually constipated, found so great difficulty in passing her stools, and how, in consequence of the close adhesions which soldered the uterus to the bladder, and of the propagation of the cancer to the anterior wall of the vagina, and perhaps to the bas-fond of the urinary reservoir, which being no longer able to undergo a sufficient dilatation, caused her to experience the continual desire to void urine? Do you not see, also, how the pains extended themselves into the left thigh in consequence of the compression and perhaps of the invasion by the same disease of the radicals of the great sciatic nerve?

I will add that, to complete the examination, we have applied the speculum, and that we have been enabled to view the vagina bristling with vegetations bleeding upon the slightest contact, and the neck of the uterus, almost entirely destroyed, converted into a fungus mass, ulcerated here and there, oozing out a fœtid ichor which bathed the entire surface. Do not forget that the employment of this latter means of diagnosis is not always applicable, and that the speculum may itself become the means of serious mischief, so that beside the pain that it occasions, it may, especially if the vagina is already invaded to a considerable extent, give rise to lacerations and hemorrhage followed by the most serious consequences. Such was the state of our patient when first examined; a few days subsequently a sypmptom presented itself which appeared extraordinary to the most of you. Some one apprised me one morning at the visit, that the patient had delirium during the night. This apyretic delirium was ever calm and tranquil—the patient uttered no cry, nor did she attempt to get up—she merely talked to herself in a low tone of voice, and did not sleep; at the same time she was restless, and had something wild in her expression of countenance. She had neither convulsions, nor coma, nor paralysis.

In the presence of this symptom, you have seen me adopt, without hesitation, the following diagnosis: *Compreesion of the ureters by the cancer, from which cause obstruction to the passage of the urine, dilatation of the ureters above the point compressed, consecutive hydronephrosis, suppression of the urinary* secretion, and, in consequence, nervous symptoms analogous to *those the authors describe under the name* URÆ-

MIA. The urine treated by heat and nitric acid, did not, however, show the presence of albumen, and percussion practiced upon the lumbar region did not reveal to me any notable augmentation in volume of the kidneys. I, nevertheless, persisted in my diagnosis, believing only that the hydronephrosis was as yet confined to narrow limits.

I was able to be so positive, because experience had many times shown me that whenever there is manifestation of delirium, or coma, or of convulsions, in the course of a cancer of the uterus, it is because there exists an obstruction to the secretion of urine, in consequence of the compression of the ureters at the point where they empty into the bladder. A little after the appearance of the delirium, which added still more to the grave state of our patient, arose a new complication which is of no less prognostic value. The 5th of May there appeared a considerable swelling of all the left inferior member, accompanied by very acute pain in the calf of the leg, a very marked elevation in the temperature of the limb, and a sensible induration, like a cord, in the direction of the femoral vessels. You will recognize here all the characteristic signs of a *phlegmasia dolens.* However, singularly enough, the symptoms rapidly amended—first the pain, and then the swelling, disappeared, and the limb had nearly subsided to its normal volume, when without any known cause as in the first instance, the œdema re-appeared in a more aggravated form throughout the same member, and invaded, also, but in a less degree, that of the right side. Now the symptoms of the double *phlegmasia dolens* continued persistently; a recto-vaginal fistula was established by reason of the incessant progress of the cancer, but the fæces were not passed by the vulva. The delirium continued in the same form free from the complication of convulsions or coma, and this wretched creature sank into an extreme state of marasmus, wasted with suffering, in spite of large doses of opiates, cherry laurel water, &c., and died the 17th of last July.

Let us now examine the lesions that the autopsy has revealed. On opening the abdomen the pelvis was found to be partly occupied by a solid mass consisting of the pelvic organs closely soldered to each other. Upon each side is to be seen mounting up towards the kidneys, the two ureters considerably distended, upon the disposition of which we will return in a moment. The hypogastric veins and the utero-ovarian plexuses are considerably distended, with black blood, and simulate the most perfect injections that we are able to make artificially. The body of the uterus is pushed backwards and to the left side, the old adhesions maintaining it fixed in this position and closely united to the other pelvic organs. The rectum has deviated from its normal direction, and carried forward by an encephaloid tumor of the size of a fœtal head, adherent to the sacrum, which is laid bare in all its left half and the sacroilliac articulation upon the same side has been invaded by the tumor. The bony structure is soft, friable, and easily penetrated by the scalpel, and the roots of the sciatic nerve, perhaps already altered in structure by the same degeneration, are subjected througout to the pressure of the tumor. Do you not understand now the irradiating pains which the patient experienced upon the posterior aspect of the thigh?

Now to the ovaries, both very small, in volume scarcely the size of an almond, they are lost in the adhesions which surround the sides of the uterus, their tissues seem to be converted into a sort of fibrous nucleus, in which we are not able to find, at least with the naked eye, any traces of their primitive organization. The fallopian tubes present no notable alteration, and seem to have escaped the cancerous degeneration.

The vagina, considerably elongated, is bathed in a fœtid ichor in which floats the organic detritus. Its anterior wall is healthy, except in the neighborhood of the cervix, where are to be found the nuclei of a substance we shall presently describe. Behind, on the contrary, the cancer occupies a great extent of surface, where is to be found the fistulous ulceration, which establishes a communication between the rectum and this canal.

The uterus, of an unusual volume, is also more than usually elevated in the pelvic cavity. That portion of the cervix which has projected into the vagina is entirely destroyed—in its place we have nothing but an ulcerated surface, irregular, manummillated, amongst the protuberences of which it is always easy to recognize an orifice which leads into the uterine cavity; this orifice, of an intense coloration, is considerably dilated, as if recently it had been distended by a liquid of which we find the traces yet remaining in a greenish yellow and fœtid sanies.

You will recall the abundant discharges of which the patient complained and the three several gushes of serous liquid that she has had suddenly, as if from the rupture of the membranes in labor, and do you not see that this liquid had no other source but the cavity of the uterus itself?

In the meantime its cavity is not regular, its posterior wall presents a convexity as large as the half of an ordinary egg; this is because the posterior wall, much hypertrophied, contains in its thickness a tumor of the volume we have mentioned, and which projects principally upon the side of the mucous membrane; its color is red and its consistence solid, but rather soft.

The absence of the pearly white color and the hardness which usually characterizes the fibrous tumors, lead us to suppose that this is encephaloid. Submitted to microscopic examination we have been unable to find anything but the elements of fibrous tissue; but not so with that of the uterus, examined principally in the vicinity of the ulceration which has eaten away the cervix, we have been enabled to establish the existence in the midst of the proper uterine fibres of masses of cells that all the microscopists regard as characteristic of cancer. They occupy the little cavities (locules) disseminated through the proper tissue of the uterus, and present the following appearance: they are voluminous, closely packed together, rounded, oval or polyphedral for the most part; some are cylindrical, others bissurcated, some have a prolongation like a tail. They are unequal in size and all enclose one or more nuclei with one or more nucleoli.

The inferior vena cava is obliterated in a great part of its course. Here then is perfectly explained the *double phlegmasia dolens.* We are obliged, however, to admit that we have not sufficiently discovered the conditions which have rendered the œdema of the right limb less considerable than that of the left side.

The large lumbar ganglia were infiltrated with cancerous material, of which we have also found two nodules from the size of a walnut to that of a hazle-nut in the parenchyma of the liver. Do not these latter alterations testify to the general injection of the economy of the cancer?

Returning now to the ureters and kidneys. These two conduits starting from the point where they enter the pelvis, in passing behind the tumor that we have described as situated behind the rectum, present a considerable dilatation, the left having attained the size of the thumb, the right that of the index finger. Their thinned walls were distended by a liquid nearly colorless or very slightly yellow. The pelves of the two kidneys had undergone a proportional development, as well as the calices, which had produced, by reason of their dilatation and the internal pressure, in the parenchyma of the kidneys a very notable atrophy of the proper tissue of these organs.

The double hydronephrosis that we have before us at this moment is not so decided as I have many times observed it. It has occurred to me, under other circumstances, to find the kidneys completely destroyed and converted into a true cyst. In such a case the kidneys attain generally a much more considerable volume, and the percussion, practiced over the lumbar region, gives an exaggerated dulness which greatly clears up the diagnosis.

If now you demand of me an explanation of this dilatation of the ureters and of the hydronephrosis, it will be easy for me to give it to you, by drawing your attention to the fact that the urine is not able to pass freely into the bladder because of the pressure exerted by the cancerous tumor of the pelvis upon these tubes, which become dilated above the point of compression, so that immediately by the accumulating urine pressure is felt upon the inner surface of the calices, and causes also atrophy of the kidneys. Along with this alteration of the venal parenchyma, the urinary secretion has been deranged, and the elimination of the residues of nutrition that these organs are charged to accomplish having become incomplete, these residues accumulating in the economy give rise to phenomena upon the part of the nervous system to which authors have given the name *uræ-*

*mia*, upon the nature and cause of which I will address you in one of our future re-unions.

If now you demand of me why the nervous phenomena were so slightly marked in our patient, since she presented but a calm delirium without convulsions or coma, I would respond that I find the reason in the limited alteration of the kidneys, which doubtless permitted these organs still to accomplish, though imperfectly, their functions. In fact, the nervous symptoms are more intense as the lesions are more profound and more extended. In support of this opinion I will limit myself to calling your attention to a single case that I observed last year at the hospital Saint-Antoine. An old cachectic woman fell suddenly upon the pavement, and was placed in my service; she had no paralysis but was in a state of profound coma. She was entirely speechless, and died in a few minutes. In this case, the suddenness of the attack, the intensity of the symptoms and the termination so promptly mortal, would make you presume that there was a considerable lesion. In fact, although we may have found at the autopsy the nervous centres perfectly healthy, the two kidneys enormously distended, so that they measure 18 to 20 centimetres (say 7½ inches) vertically, were almost literally converted into two cysts—so much so that there remained little of the renal substance, which had been destroyed, as in the case of to-day, by the accumulation of urine in the ureters compressed and obliterated by a cancer of the uterus.

